# Technologies for the Synthesis of mRNA-Encoding Libraries and Discovery of Bioactive Natural Product-Inspired Non-Traditional Macrocyclic Peptides

**DOI:** 10.3390/molecules18033502

**Published:** 2013-03-18

**Authors:** Kenichiro Ito, Toby Passioura, Hiroaki Suga

**Affiliations:** Department of Chemistry, Graduate School of Science, The University of Tokyo, 7-3-1 Hongo, Bunkyo-ku, Tokyo 113-0033, Japan; E-Mails: k-ito@chem.s.u-tokyo.ac.jp (K.I.); toby@chem.s.u-tokyo.ac.jp (T.P.)

**Keywords:** non-standard macrocyclic peptides, non-canonical amino acids, flexizyme, Random non-standard Peptide Integration Discovery (RaPID) system, peptide inhibitors

## Abstract

In this review, we discuss emerging technologies for drug discovery, which yields novel molecular scaffolds based on natural product-inspired non-traditional peptides expressed using the translation machinery. Unlike natural products, these technologies allow for constructing mRNA-encoding libraries of macrocyclic peptides containing non-canonical sidechains and N-methyl-modified backbones. The complexity of sequence space in such libraries reaches as high as a trillion (>10^12^), affording initial hits of high affinity ligands against protein targets. Although this article comprehensively covers several related technologies, we discuss in greater detail the technical development and advantages of the Random non-standard Peptide Integration Discovery (RaPID) system, including the recent identification of inhibitors against various therapeutic targets.

## 1. Introduction

In the contemporary pharmaceutical industry, the dominant paradigm of drug discovery relies on the identification of bioactive low molecular weight chemical compounds (ideally less than 500 Da according to the Lipinski “rule of five” criterion) by high-throughput screening of synthetic or natural product chemical libraries. “Hits” from these screens are then advanced through a “hit to lead” process to generate lead compounds for pre-clinical and clinical evaluation. Whilst this approach has proven successful for the discovery of a number of blockbuster drugs, there has been a steady decline in the number of new molecular entities (drugs containing novel core structural scaffolds, as opposed to new drugs which are analogues of existing therapeutics) developed since the mid 1990s [1]. To some extent, the increasing clinical use of biologics (antibodies or proteins) has made up for the decreasing rate of discovery of novel small molecule drugs. For example, the monoclonal antibody infliximab (trade name Remicade™, Janssen) is now one of the most highly prescribed medicines in the World. However, a major shortcoming of biologics is that due to their large molecular weight, ideal pharmacokinetic properties such as membrane-permeability and oral-bioavailability have proven difficult to achieve. Consequently, biologics generally require parenteral administration and their therapeutic targets are restricted to the extracellular milieu. Thus, molecular scaffolds that gain the advantages of small molecule drugs and biologics but reduce or omit their disadvantages are ideal for the development of drugs.

As an alternative to both low molecular weight compounds and large biologics, peptides represent an attractive scaffold for the development of novel classes of drugs [2]. Although peptides are a well established molecular scaffold, recent descriptions of “non-traditional” peptide scaffolds, including stapled α-helices and various macrocyclic structures, have revived interest in peptide drugs [3]. In particular, “medium sized” (molecular weights in the range of 1,000–3,000 Da), macrocyclic, *N*-methylated peptides appear to combine high bioactivity with desirable pharmacokinetic properties. This is because macrocyclization improves membrane permeability by both removing the charged termini from the peptide and facilitating intramolecular hydrogen bonding, whilst *N*-methylation also improves membrane permeability by decreasing the potential for intermolecular hydrogen bonding [4,5,6]. Further, both modifications grant some degree of resistance to proteases [7,8]. Novel technologies facilitating the identification of such peptides have tremendous potential to revolutionize the speed of drug discovery. Moreover, such technologies have the potential to produce highly potent and selective drugs against a wide range of extra- and intra-cellular targets that have not been amenable to targeting with small molecules. The present review focuses on emerging technologies for the discovery of macrocyclic peptides against various therapeutic targets from mRNA-encoding random peptide libraries.

## 2. Naturally Occurring Peptides and Synthetic Natural Product-Inspired Peptides

Naturally occurring peptides can be classified into two general classes. The first class includes “standard” peptides, which are linear molecules comprised of residues of the 20 canonical amino acids. These are typically generated via ribosomal synthesis and may be post-translationally modified by ligation to carbohydrates or lipids, or through cleavage by a specific protease(s). Well-studied examples include peptide hormones such as insulin and glucagon-like peptide-1 (GLP-1). It should be noted that these peptides consist only of proteinogenic amino acids, although in some instances the sidechains may be post-translationally modified with certain naturally occurring chemical groups. 

**Figure 1:**
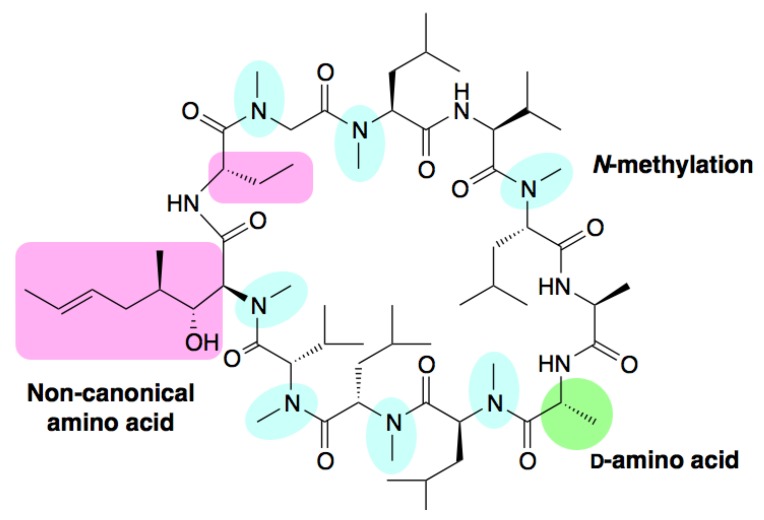
Structural characteristics of cyclosporine A. Cyclosporin A is an 11 residue N-C macrocyclic peptide. Seven peptide bonds are *N-*methylated (highlighted in blue). Two residues possess non-proteinogenic sidechains (highlighted in pink) and a single d-alanine is present (highlighted in green) [11].

The peptides in the second class contain non-proteinogenic amino acids, such as l-amino acids containing non-canonical sidechains, d-amino acids, and *N-*methyl modifications. The “non-standard” peptides in this class are often found as secondary metabolites of bacteria, fungi, and other microorganisms, and have anti-microbial properties that provide an evolutionary advantage to the species that produce them. Consequently, many of these compounds have found extensive clinical use as antibiotics. In Nature, some of these peptides are expressed by the translation machinery similar to the “standard” peptides discussed above, but extensively modified by post-translational modifying enzymes (such as heterocyclases and macrocyclases) to produce the mature peptide(s) [9]. However, many of the peptides in this class are synthesized *de novo* by the action of non-ribosomal peptide synthetases (NRPSs) [10,11]. One such compound is the drug cyclosporin A (Figure 1). Originally isolated from the fungus *Tolypocladim inflatum*, cyclosporin A is an orally-bioavailable immunosuppressant used for the treatment of certain autoimmune diseases and in organ transplantation settings. It is remarkable that cyclosporin A bears a highly modified peptide scaffold including seven backbone *N-*methylations, two residues with non-canonical sidechains, a macrocyclic backbone and a d-alanine residue. These unique features are essential for cyclosporine A’s drug-like properties; *i.e.*, cell membrane permeability, serum stability (resistance to proteases and peptidases) and oral bioavailability even though cyclosporin A violates two of the Lipinski rules (a molecular weight substantially greater than 500 Da and greater than 10 hydrogen bond acceptors).

Although this second class of “non-standard” natural product peptides potentially provide excellent scaffolds for the development of novel drugs, the structural and mechanistic complexities of NRPS gene clusters have greatly hampered efforts to engineer them for the production of *de novo* chemical libraries [12,13,14]. For this reason, to the best of our knowledge no peptide with activity against a novel therapeutic target has yet been generated through the reengineering of NRPSs. More recently, however, alternative approaches for the production of natural product-inspired non-standard peptides using ribosomal translation have been devised to achieve a similar goal. This review discusses such emerging technologies.

## 3. Flexizymes and the FIT System: Tools for Genetic Code Reprogramming and the Rapid Synthesis of Natural Product-inspired Non-standard Peptides

**Figure 2:**
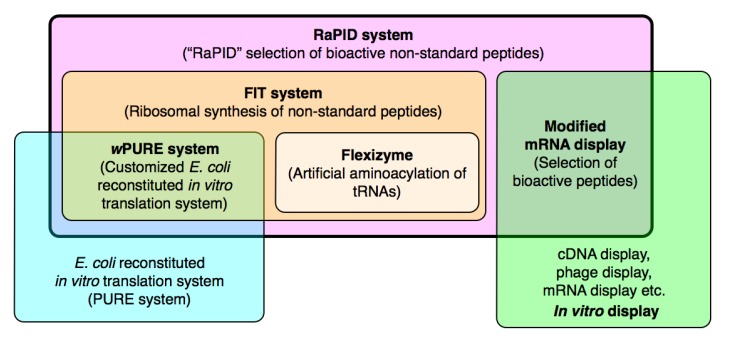
Overview of the tools discussed in this review. The RaPID system (pink), which is the primary tool for drug-like peptide discovery discussed in this review, is derived from a combination of the FIT system (orange) with a modified mRNA display technique (green) [15,16]. The FIT system itself is derived from a combination of a modified PURE *in vitro* translation system (blue) [17] with flexizyme technology (tan) [18,19,20,21,22,23,24,25,26,27]. Acronyms are defined in the text.

This section describes how the tools that facilitate the genetic code reprogramming and rapid synthesis of non-standard peptides using the translation apparatus were developed. An overview of the technologies discussed in this, and the following, section is shown in Figure 2.

### 3.1. Limitations of the Natural Genetic Code and Use of Expanded Genetic Codes: Towards Genetic Reprograming

*In vivo*, the translation machinery uses the genetic code in which each of the 20 proteinogenic amino acids are assigned to specific codons, and synthesizes peptide chains according to the sequence of an mRNA template. In consequence, engineering of the peptide sequence can be achieved in a facile manner by altering the mRNA template (or its cognate DNA), a feature that distinguishes ribosomal peptide syntheses from NRPS mediated peptide synthesis. However, the restriction of the genetic code means that only peptides consisting of the 20 proteinogenic amino acids can be synthesized ribosomally. A classical strategy to generate peptides (proteins) incorporating one or two non-proteinogenic amino acids is to use triplet stop codons (non-sense codons) [28] or quadruplet codons [29] that are suppressed with tRNAs charged by engineered aminoacyl-tRNA synthetases (ARSs) *in vivo*. Despite a number of elegant studies performed using this methodology, it lacks sufficient flexibility to generate diverse non-standard peptides containing the more exotic non-proteinogenic amino acids, such as *N-*methyl-amino acids or d-amino acids, at the aforementioned level of structural complexity exhibited by natural products such as cyclosporin A. 

The cell-free (*in vitro*) translation approach is a more flexible and controllable system for the expression of such non-standard peptides. Even so, to achieve this goal it remains critical to develop appropriate tools for charging a wide array of non-proteinogenic amino acids onto tRNAs, making vacant codons in the genetic code, and then reassigning the non-proteinogenic amino acids with the non-proteinogenic aminoacyl-tRNAs. This strategy is referred to as genetic code “reprogramming”, as opposed to genetic code expansion.

### 3.2. Flexizymes

Although the concept of genetic code reprogramming is simple, critical tools are required for its realization. First a technique is required to prepare non-proteinogenic aminoacyl-tRNAs in a reliable and flexible manner. At the same time, any residual uncharged tRNA must not be recharged by the endogenous aminoacyl-tRNA synthetases in the translation system. Although a semi-synthetic method for the preparation of non-proteinogenic aminoacyl-tRNAs has been used in the aforementioned *in vitro* genetic code expansion, it is technically demanding due to the requirement for purification and deprotection of the chemical intermediates [30], making this strategy difficult for most researchers to pursue. As an alternative, we have devised a catalytic system, based on aminoacylating ribozymes known as flexizymes, in order to facilitate this critical step.

The evolutionary history of flexizymes has been reviewed recently [18,19,20,21,22,23,24,25,26,27] and is beyond the scope of this review, however the characteristics of the three most useful flexizymes will be briefly discussed here. The term flexizyme is derived from flexible tRNA acylation ribozyme. Each flexizyme consists of 45 or 46 nucleotides (nt), the 3′-trinucleotide motif of which hybridizes with the single strand region of the tRNA 3′-terminus. Because of the strong base pair interaction between a GG dinucleotide in the flexizyme and a CC dinucleotide that is conserved in all tRNAs, and a weak interaction between a U in the flexizyme and the tRNA’s discriminator base (A, G, and U, but C is also tolerated at a high Mg^2+^ concentration), virtually any tRNA will act as a flexizyme substrate [31]. 

The three main flexizymes are: dinitroflexizyme (dFx), enhanced flexizyme (eFx) and amino- flexizyme (aFx). dFx uses any acid esterified with a 3,5-dinitrobenzyl (DNB) group as a substrate, and thus acts as a generic acylation catalyst for most acyl-donor substrates independent of the sidechain structure. The substrates for eFx [32] are aromatic amino acids esterified with cyanomethyl (CME) groups or acids esterified with a *p-*chlorobenzyl thioester (CBT) moiety independent of the sidechain structure. aFx [33] acts on amino acids esterified with an amino-modified benzylthiol (ABT), and is of particular use when esterification of the amino acid with DNB causes poor solubility in the reaction buffer (only an issue with highly hydrophobic sidechains). In this context, the protonated amino group on ABT improves the water solubility. Through the use of these three flexizymes, an extremely wide range of organic acids including α-amino acids with non-canonical sidechains, *N-*alkyl-α-amino acids [34,35], *N-*acyl-α-amino acids [36], d-α-amino acids [37], polypeptides [38] and α-hydroxyl acids [39] can be charged onto virtually any tRNAs independent of body or anticodon sequence.

### 3.3. Orthogonal tRNAs

The ribosome will accept as substrates a wide range of pre-charged non-proteinogenic aminoacyl-tRNAs. In practice, however, deacylated tRNAs would be expected to be generated during the translation reaction either by release from the ribosome after elongation or simply through background hydrolysis of the non-proteinogenic aminoacyl-tRNAs. These deacylated tRNAs would then serve as substrates for endogenous ARSs in the translation system, and would thus be charged with their cognate proteinogenic amino acids. The resulting “mischarged” tRNAs would potentially suppress incorporation of the desired non-proteinogenic amino acids leading to native background in the reprogrammed genetic code. To avoid this, tRNAs must be engineered to be inert with respect to endogenous ARSs. Such tRNAs have been termed orthogonal tRNAs [40].

Although there have been several orthogonal tRNAs reported in literature, we have predominantly employed one particular tRNA body sequence originating from tRNA^Asn^. This tRNA body sequence, refered to as tRNA^Asn–E1^ [39], was originally derived from the amber suppressor tRNA^Asn^_CUA_ [41], but during the course of our attempts to devise a universal method of genetic code reprogramming we found that various anticodons could be implanted in this tRNA without affecting its inertness against ARSs. Therefore, the use of this single species of tRNA has become the platform for the preparation of desired orthogonal tRNAs bearing various anticodons in our laboratory.

### 3.4. Genetic Code Reprogramming in the FIT System

**Figure 3:**
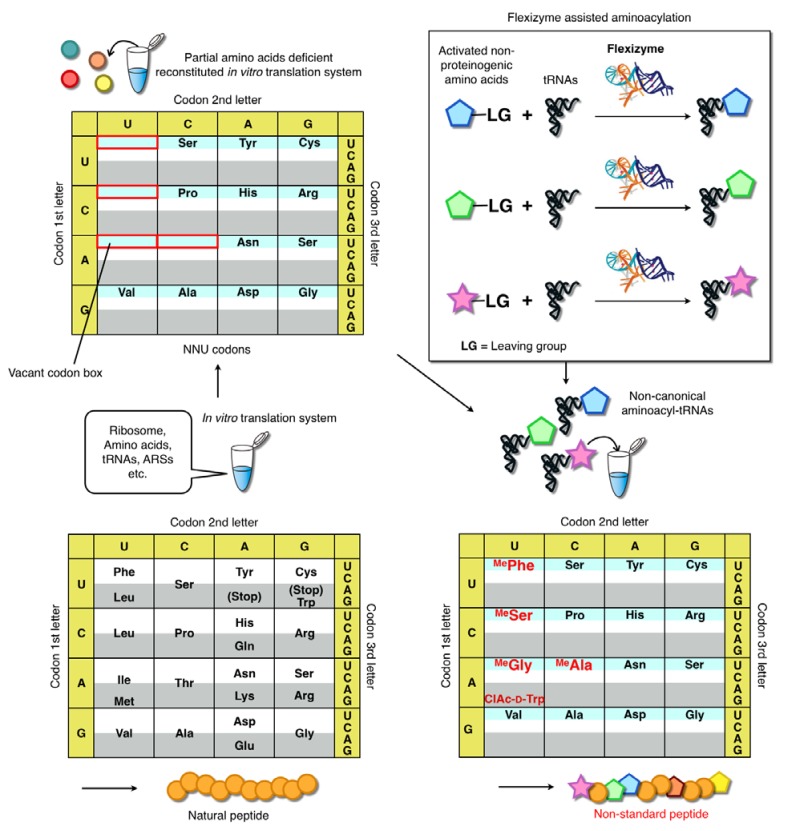
Genetic code reprogramming using the FIT system [22]. Peptides are ribosomally synthesized in this system through the activity of a fully reconstituted *in vitro* translation reaction. Amino acids or other factors can be omitted from the reaction as desired. The figure depicts a reaction in which 5 canonical amino acids (Phe, Leu, Ile, Thr and Met) have been omitted from the system to create the vacant codons. Addition of the corresponding aminoacyl-tRNAs charged with non-canonical amino acid by flexizymes (in this instance chloroacetyl-d-tryptophane: ClAc-d-Trp and four *N-*methyl amino acids: ^Me^X) enables for “reprogramming” of codons to ribosomally synthesize peptides that contain the designated non-canonical amino acids.

It can be seen that flexizyme technology readily allows the preparation of non-proteinogenic aminoacyl-tRNAs. However, to use these aminoacyl-tRNAs for genetic code reprograming requires the creation of “vacant” codon boxes in the genetic code that can be “filled” with the desired non-proteinogenic amino acids. To achieve this, a modified version of the *E. coli* based *in vitro* reconstituted translation system described by Shimizu *et al.* has been employed. The original system, known as the PURE (protein synthesis using reconstituted elements) system, is entirely composed of defined elements [17]. Importantly, it is readily customizable since any given translation factor, enzyme or amino acid can be omitted. If specific amino acids and/or their cognate ARSs are not included in the reconstituted system, the codons encoding the omitted amino acids become vacant in the codon table (Figure 3). 

These vacant codons can be filled by the introduction of non-proteinogenic aminoacyl-tRNAs prepared using flexizymes, thus achieving the assignment of the desired amino acids to the desired codons. Moreover, other translation factors such as methionine-tRNA formyltransferase (MTF), release factors (RFs), ribosome recycling factors (RRF), and others can be omitted from the reaction as required for specific purposes. Early versions of this customized system were known as the “withdrawn PURE system”. However, more recent studies have employed a range of different custom translation systems depending on the applications. Examples include the addition of enzymes not included in the original PURE system (e.g., enzymes that remove formyl-methinone) [42] or the alteration of protein factor concentrations to elevate the level of non-proteinogenic amino acid elongations for the expression of exotic short peptides. Thus, we renamed this general methodology the Flexible In-vitro Translation (FIT) system. The use of this system enables us to perform extensive genetic code reprogramming, and thus to devise strategies for expressing libraries of non-standard peptides inspired by natural products.

### 3.5. Ribosomal Synthesis of Biopolymers in the FIT System

The first demonstration of non-standard peptide synthesis using a reprogrammed codon box was performed by Forster and coworkers [43]. This team reset the elongation codon box and refilled it with three non-proteinogenic amino acids by reconstituting the essential components of translation from *E. coli* (70S ribosome, initiation factors, elongation factors, fMet-tRNA^fMet^_CAU_) and non-proteinogenic aminoacyl-tRNAs prepared by the classical semi-enzymatic chemical synthesis method. This system demonstrated the potential for ribosomal expression of artificial peptides using a reconstituted translation system. However, because this reaction system did not contain release factors and ribosomal recycling factors, it was limited to single turnover synthesis and thus the achievable peptide yield was relatively low. Additionally, since the codon table contained only initiator methionine and three artificial amino acids for elongation (*i.e.*, all other codon boxes remained vacant) it remained unclear whether filling these empty codon boxes (with other proteinogenic or non-proteinogenic amino acids) would allow expression of more diverse peptides with sufficient fidelity. In consequence, these issues had to be addressed in order to fully realize the potential of ribosomal synthesis of non-standard peptides under a reprogrammed genetic code.

We sought to take advantage of the FIT system to not only resolve these issues but also to extend genetic code reprogramming to the synthesis of other biopolymers. In 1970, Fahnestock and Rich published the landmark finding that the ribosome is capable of polymerizing the non-α-amino acid substrate, phenyllactic acid (F^lac^) [44]. Although this demonstration did not directly confirm the production of the resulting polyester (which was most likely a heterogeneous mix of F and F^lac^) due to the limitations of the analytical methods available at the time, indirect data confirmed that ester bond formation was catalyzed by the ribosome. In light of this finding, we decided to investigate ribosomal synthesis of polyester-polypeptide hybrids using the FIT system. The findings published in 2007 [39] demonstrated that up to seven different α-hydroxy acids could be assigned to the genetic code, and diverse polyesters of up to 12 consecutive residues followed by a Flag-tag peptide could be expressed using the FIT system. The genetic code created in this work assigned seven different hydroxy acids and four amino acids encoding the Flag peptide. Most importantly, we were able to detect the polyester-polypeptide hybrids by polyacrylamide gel electrophoresis, and also to directly confirm the sequence of a section of the polyester region (up to 4-mer) by mass spectrometry. This represented an important step with respect to novel biopolymer synthesis using a reprogrammed genetic code.

The following year, we reported ribosomal synthesis of *N-*methyl peptides using the FIT system. In that study, we examined the complete set of *N-*methyl derivatives of the proteinogenic α-amino acids, revealing which could be efficiently incorporated into peptide chains. We were able to demonstrate that up to 10 consecutive *N-*methyl-α-amino acids could be polymerized using the FIT system. Moreover, by integrating *N-*methyl-peptide synthesis with thioether macrocyclization (*vide infra*) [35], we showed a potential route to the construction of natural product-like macrocyclic *N-*methyl-peptides with specified sequences.

This methodology was further extended to *N-*alkyl-glycines, yielding polypeptoids and peptoid-peptide hybrids [34]. We found that *N-*modification could be varied from simple hydrocarbon chains to those with bioorthogonal functional moieties (hydroxy, cyano, allyl, alkyne, and azide groups). As with the *N-*methyl peptides discussed above, this methodology could be integrated with a macrocyclization method to yield macrocyclic peptoid-peptide hybrid molecules, an attractive scaffold for the discovery of novel bioactive peptidomimetics.

### 3.6. Macrocyclization Methodologies Compatible with the FIT System

Bioactive natural product peptides often exhibit macrocyclic structures. The conformational constraints that this imbues contribute to their physiological stability as well as their high affinity to interacting partner proteins. It seemed critical, therefore, to devise methodologies that enable the macrocyclization of linear peptides encoded by mRNA templates. The flexibility of the FIT system allows for the incorporation of diverse non-proteinogenic amino acids that can be used to direct macrocyclization in a manner compatible with the translation apparatus. Indeed, as discussed below, we have devised several methodologies and demonstrated macrocyclization of peptides expressed in the appropriate FIT system.

Thioether macrocyclization, which is typically conducted by the reaction between a 2-haloacetyl group and the thiol moiety in a cysteine (Cys) sidechain, is a classical methodology for peptides prepared by standard chemical synthesis. However, this methodology had never been integrated with ribosomal peptide synthesis. The conditions required for macrocyclization during chemical synthesis and ribosomal synthesis are quite distinct from each other. The former generally uses an organic solvent with a relatively high peptide concentration (mM range), whereas the latter must use an aqueous buffer containing various nucleophilic thiols, (e.g., Cys and reducing agents such as mercaptoethanol and dithiothreitol) at a relatively dilute peptide concentration (µM range).

**Figure 4:**
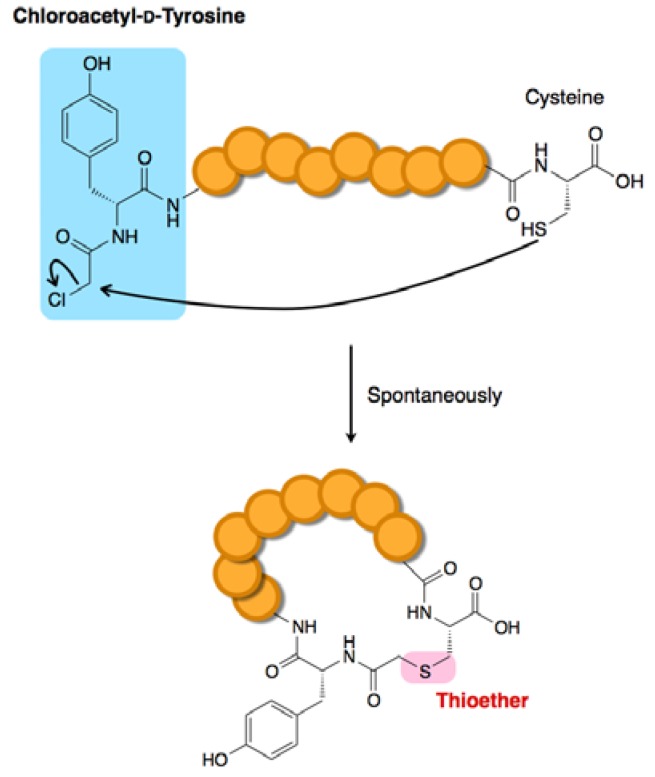
Macrocyclization by post-translational thioether-ring closure [36,45]. A chloroacetyl-amino acid incorporated at the *N*-terminus using the FIT system spontaneously reacts with a downstream Cys to generate a macrocyclic thioether bond.

Prior to the development of thioether macrocyclization methodologies, we had devised a method for initiation codon reprogramming using a methionine-deficient FIT system, in which the vacant initiator codon was reassigned to various α-l/d-amino acids and their acyl-derivatives. This method then served as the foundation for various macrocyclization methodologies. Thioether macrocyclization of chemically synthesized peptides is typically achieved through the use of a 2-bromoacetyl group. However, we found that incorporation of this group at the *N*-terminus of peptides was incompatible with the translation system due to the formation of undesired thioether adducts originating from rapid intermolecular reactions between the aforementioned thiol reagents and the 2-bromoacetyl group. By contrast, a 2-choloroacetyl group incorporated at the *N*-terminus turned out to be highly selective for intramolecular thioether bond formation with a downstream Cys. In this method, an *N-*2-chloroacetyl-α-amino acid (e.g., *N-*2-chloroacetyl-tyrosine: ClAc-d-Tyr—shown in Figure 4) is assigned to the initiator codon and peptides bearing a Cys residue(s) at a downstream position(s) can be expressed. Under these conditions, the thioether macrocyclization occurs spontaneously without additional reagents, yielding the desired macrocyclic peptide without side products originating from intermolecular reactions. This high selectivity for the intramolecular reaction may result from the high dilution factor of the expressed peptides ranging from sub-µM to 10 µM; however, an alternative possibility, which has not been completely ruled out, is that the ribosome might protect against such undesired intermolecular reactions and intramolecular macrocyclization of the peptide chain may even occur in the ribosome tunnel. 

**Figure 5:**
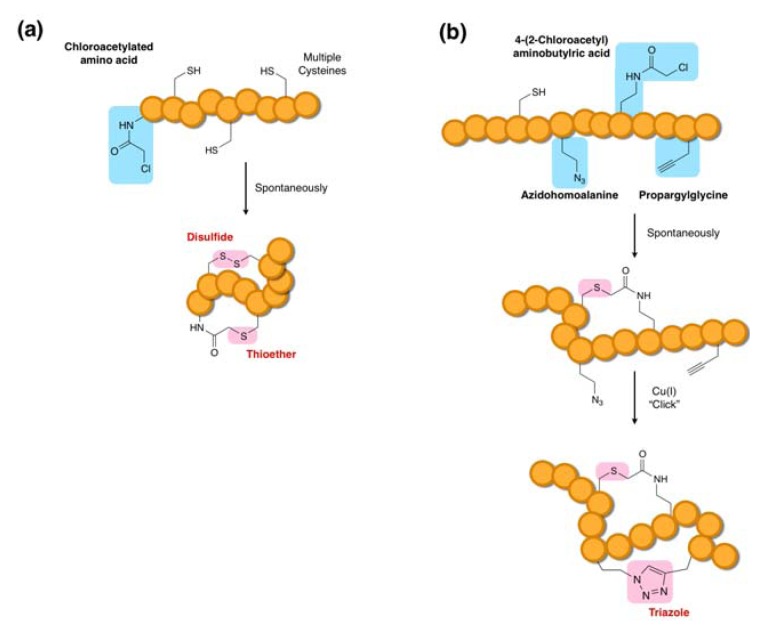
Ribosomal synthesis of bicyclic peptides. (**a**) Synthesis of an overlapped bicyclic peptide coupled with thioether and disulfide bond formations [45]. (**b**) Synthesis of a bicyclic peptide coupled with post-translational ring closure by a “click” cycloaddition reaction. The azide group of azidohomoalanine reacts with the alkyne group of propargylglycine to form a triazole moiety in a reaction catalyzed by Cu(I) [46].

Through extensive studies, we have found that this particular thioether macrocyclization chemistry designed into the FIT system occurs regardless of peptide sequence composition and length. There is, however, one exception; a Cys incorporated at the second position (note that the first position is the initiating amino acid residue that attached to the ClAc group) will not react with the chloroacetyl moiety, leaving this Cys residue intact [45]. This observation turns out to be useful for the design of bicyclic peptides in which the first Cys is incorporated at the second position and the second and third Cys residues are incorporated in downstream positions. In such a construct, the second Cys residue selectively reacts with the N-terminal ClAc group and the intact first and third Cys residues form a disulfide linkage, yielding an overlapping bicyclic peptide scaffold. In this manner, various compact structures of overlapping bicyclic peptides closed by a thioether bond and a disulfide bond can be expressed as designed (Figure 5a).

Another strategy generating an overlapping bicyclic scaffold has been devised based on the Cu(I)-catalyzed alkyne–azide cycloaddition reaction, known as “click” chemistry (Figure 5b) in combination with the thioether macrocyclization method [46]. In this case, the FIT system was used to incorporate azidohomoalanine (Aha) and propargylglycine (Pgl) into peptides and the post-translational treatment of the peptides with Cu(I) as a catalyst resulted in a triazole formation. The orthogonality of the click macrocyclization to the thioether macrocyclization enables us to selectively form thioether bonds spontaneously upon translation and then form the triazole bond through treatment with the catalyst. The advantage of this methodology over the aforementioned thioether-disulfide bond formation methodology is that any positions can be chosen for bicycliczation, so that diverse bicyclic structures can be generated. Moreover, both macrocyclic linkages in the latter methodology are non-reducible and therefore physiologically more robust, whereas the former contains a reducible disulfide bond. On the other hand, the latter methodology requires post-translational conversion of the peptides leading to incomplete macrocyclization, whereas the former methodology auto-bicyclizes the expressed peptides in a nearly quantitative manner. Because of the simplicity of the procedure, the former methodology may be more readily adoptable to selection methods (*vide infra*).

**Figure 6:**
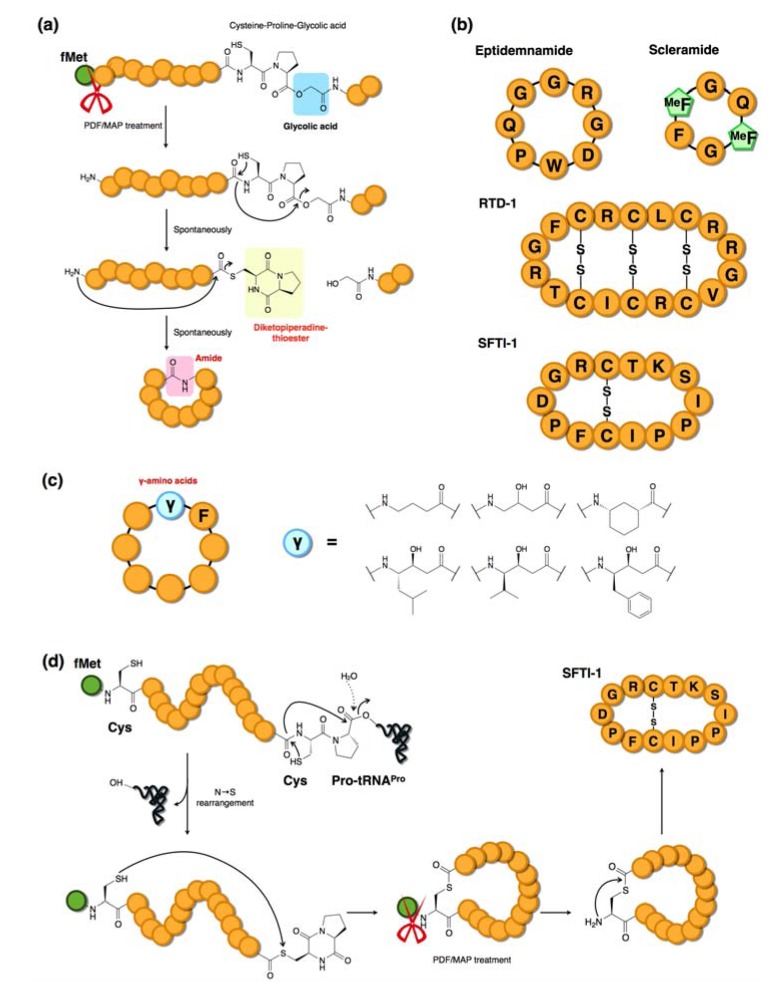
Mainchain macrocyclizations via diketopiperadine (dkp) formation. (**a**) Incorporation of a glycolic acid residue at the downstream from a Cys and Pro leads to spontaneous formation of a dkp-thioester. The N-terminal amine generated by enzymes (PDF/MAP) attacks the thioester and the mainchain is closed by the newly formed amide bond [42]. (**b**) Four mainchain macrocyclized peptides produced by the FIT system [42,48]. (**c**) Various γ-amino acid-containing macrocyclic peptides synthesized by mainchain macrocyclization [48]. (**d**) Promotion of macrocyclization by the ribosome drop-off event of peptidyl-tRNA [49].

The FIT system has also been used to synthesize naturally occurring backbone mainchain-cyclized peptides in which N-terminus to C-terminus macrocyclization occurs via the formation of a peptide bond [42]. To achieve this, we adopted a methodology originally devised by Aimoto *et al.* who demonstrated the formation of a C-terminal diketopiperadine-thioester (dkp-thioester) via a Cys-Pro-ester generated by chemical synthesis [47]. We expressed a peptide sequence with the structure: peptide-Cys-Pro-^HO^G where ^HO^G (glycolic acid) was assigned by a reprogrammed codon in the FIT system. Moreover, this particular FIT system included two N-terminal modification enzymes, peptide deformylase (PDF) and methionine aminopeptidease (MAP), which catalyzed the removal of formyl-methionine (fMet) to liberate a free amino group at the N-terminus. Thus, the Cys-Pro-^HO^G residues self-rearranged to a dkp-thioester derived from the conjugation of C and P via the non-enzymatic N→S equilibrium shift trapped by cleavage at the ^HO^G residue (Figure 6a). The resulting thioester is then attacked by the N-terminal amino group liberated by the PDF/MAP enzymatic removal of the fMet group, thus forming a backbone macrocyclic structure closed at the N- and C-termini. Using this technology, we have demonstrated the synthesis of four naturally occurring cyclic peptides (Figure 6b), eptidemnamide, scleramide, rhesus theta-defensin-1 (RTD-1), sunflower trypsin inhibitor-1 (SFTI-1) and their analogs using a reprogrammed genetic code [42,48]. This method was further extended by integration with initiation reprogramming using γ-amino acids and applied to the synthesis of γ-amino acid-containing macrocyclic peptides (Figure 6c) [48]. More recently, the same C-terminal dkp-thioester was generated through a ribosome drop-off event (the ester bond between the peptidyl group and the 3′-OH of the tRNA adenosine was analogous to the ester bond of Cys-Pro-^HO^G) to promote macrocyclization (Figure 6d) [49].

The examples discussed above have demonstrated that the combination of the FIT system with various macrocyclization strategies can produce a diverse array of natural and unnatural macrocyclic peptide structures. Such structural variations of the peptide backbones, in conjunction with the diverse residues afforded by the FIT system, are major advantages in the production of natural product-inspired peptides and peptidomimetics.

## 4. The RaPID System: An Enabling Technology for the Rapid Discovery of Natural Product-inspired Bioactive Peptides

**Figure 7:**
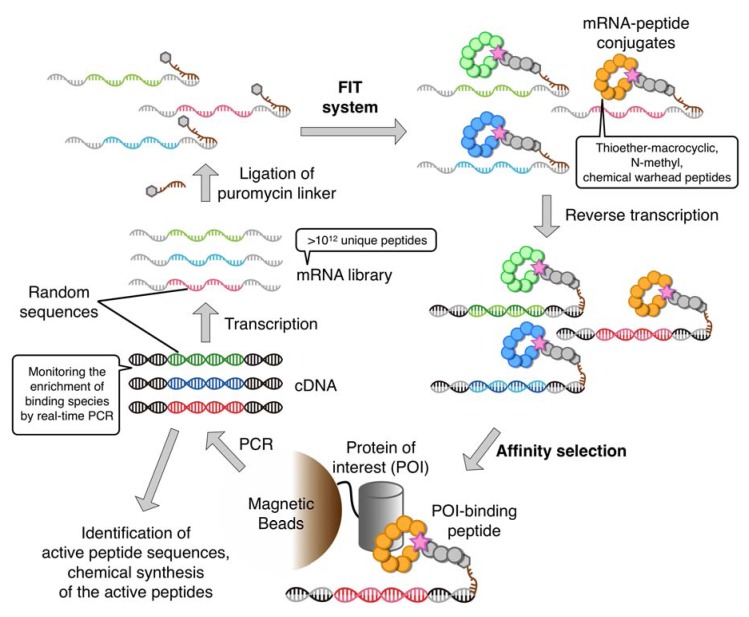
Schematic illustration of the RaPID system. First, a non-standard macrocyclic peptide library is ribosomally synthesized from an initial random mRNA library, each of which peptide is linked to its cognate cDNA by ligation of the mRNA to the peptide via a puromycin linker. After reverse transcription, peptides that potentially bind to the target protein are then isolated by an appropriate affinity-based selection procedure. cDNAs encoding the “hit” peptides are amplified and transcribed to generate the next, enriched, library.

The FIT system readily allows for the rapid preparation of a wide variety of non-standard peptides (*i.e.*, chemical libraries) from their respective oligonucleotide templates. In order to identify bioactive non-standard peptides from such libraries, the FIT system needs to be integrated with an *in vitro* display method. Although there are several *in vitro* display techniques available (e.g*.*, ribosome and DNA displays [50,51,52]), our choice was the puromycin linker-based mRNA display technique originally devised independently by the Szostak group and Yanagawa group (who termed it *in vitro* virus) [15,16]. This was because (1) it forms a covalent linkage between phenotype (non-standard peptide) and its encoding genotype (mRNA) facilitating manipulation of the selection conditions, and (2) it is compatible with higher library complexities (>10^12^) than other display techniques. We believed that the customizability of the FIT system combined with the technical simplicity of mRNA display was the best match for the construction of a new enabling technology for the discovery of bioactive natural product-like non-standard peptides. We thus referred to this combined system as the Random non-standard Peptides Integrated Discovery (RaPID) system (Figure 7).

Although several molecular designs are feasible for the puromycin-linker [53,54,55], we chose the simplest design, consisting of a 3′-puromycin-CC-PEG-DNA oligonucleotide, the DNA component of which is designed such that it is complementary to the 3′-terminus of the mRNA to which it is to be ligated. Leaving a few unpaired bases at the 3′-terminus of the mRNA (*i.e.*, the 5′-terminus of the DNA oligonucleotide) allows the formation of a small loop that can be enzymatically ligated using T4 RNA ligase. Such a strategy has the advantages of reliability and economy, and upon optimizing the annealing sequence, length, and junction loop sequence, ligation can be achieved in a nearly quantitative manner. Following translation of the mRNA, the puromycin moiety is covalently linked to the newly translated mRNA through the catalytic activity of the ribosome, creating the covalently linked peptide-mRNA fusion. Alternatively, a 3′-ACC-PEG-DNA oligonucleotide charged with Phe by eFx (3′-Phe-ACC-PEG-DNA) and a non-ligated duplex with mRNA can be utilized (PeptiDream Inc., personal communication).

A typical mRNA library for the display of thioether macrocyclic peptides can be constructed as follows: A random sequence region is embedded between the initiation codon (AUG) and the Cys codon (UGU) followed by a (GGC-AGC)_3_ motif encoding (Gly-Ser)_3_ and a UAG stop codon. The sequence downstream of the peptide-coding region is designed to have a G-rich short nucleotide sequence that anneals to the complementary region of DNA in the aforementioned puromycin linker, and the unpaired sequence regions are ligated to form a tri-nucleotide loop. It should be noted that the random sequence region generally consists of NNK repeats where N is U, C, A, or G, while K represents U or G. Depending on the purpose of the selection, NNU or NNC repeats have also been used (*vide infra*). Moreover, the number of repeats can be varied from 5 to 15, and a mixture of the respective lengths of peptide-encoding mRNA libraries is generally prepared for the subsequent translation.

This library is then translated in the FIT system, which allows for reprogramming of the initiation codon to a non-canonical ClAc-amino acid such as ClAc-d-Tyr. The Cys residue incorporated downstream of the random sequence region (or a Cys residue contained in the random sequence) reacts with the N-terminal ClAc group to form a thioether bond, yielding thioether-macrocyclic peptides after the completion of translation. When the ribosome reaches the UAG stop codon, it stalls due to the lack of RF1 (the release factor for the UAG stop codon). The α-amino group on puromycin (or Phe-adenosine) is ligated to the C-terminus of the peptide chain through the catalytic activity of the ribosome, forming a covalent link between the newly synthesized peptide and its cognate mRNA. The use of the RF1-deficient FIT system avoids undesired termination and enhances the efficiency of the peptide-mRNA fusion. This “tagging” reaction gives a 1:1 correspondence between each thioether-macrocyclic peptide and its encoding mRNA. Subsequently, the mRNA template region of the peptide-mRNA fusion is reverse-transcribed to cDNA to form an mRNA-cDNA hybrid duplex.

The above library of peptide-mRNA•cDNA fusions is then applied to a target protein of interest immobilized on magnetic beads (or resin) via an appropriate purification tag, e.g., biotin tag–streptavidin beads. The library is first subjected to a background subtraction by incubating with the parental beads, and flow through is collected. Repeating this process several times depending on the selection, the flow through library is then applied to the protein-beads to isolate active peptide binders. The selected cDNAs of the fusions are amplified by PCR, transcribed into an enriched mRNA library, and the selection cycle is repeated under elevating selective pressure whilst monitoring the enrichment of binding species using real-time PCR. The enriched pools are then cloned and subjected to DNA sequencing to determine the active peptide sequences. Alignment of these sequences reveals the evolutionary convergence of active peptides. Several clones are chosen for the preparation of the respective peptide-mRNA•cDNA fusions, and the respective clones are assayed for binding to the target protein on beads to confirm binding. We then chemically synthesize each peptide for further studies of binding affinity and bioactivity.

One round of the selection and enrichment in the RaPID system can be completed in a day or less. This rapidity is superior to the original mRNA display (taking several days to a few weeks [56]) and other screening systems such as phage display [57] or encoded combinatorial chemical library screening [58]. The complexity of the initial RaPID library is greater than a trillion sequences (>10^12^), which is orders of magnitude greater than comparable screening systems. Although the RaPID system described in the example above only allows for the selection from thioether-macrocyclic peptide libraries, customized FIT systems that enable the expression of *N-*methyl-peptide libraries or those including various chemical warheads can be applied to RaPID selection. Therefore, not only the sequence space of peptide libraries but also the diversity of chemical entities included in the peptide residues is practically infinite. In the next section, a few examples of published work are briefly discussed although many other examples will be published in the near future.

## 5. Bioactive Non-Standard Thioether-Macrocyclic Peptides Identified by the RaPID System

### 5.1. AKT2-Isoform Selective Inhibitors

Members of the human AKT serine/threonine (S/T) kinase family play critical roles in intracellular signal transduction [59]. Misregulation of AKT signaling affects apoptotic mechanisms, cellular proliferation and metabolism, and AKT inhibitors are, therefore, potentially useful as therapeutics. Of the three human AKT isoforms (AKT1, AKT2, and AKT3), it has been suggested that AKT2 plays a specific role in insulin receptor signal transduction, implying that AKT2 may be a useful target for the treatment of diabetes mellitus. Because the other isoforms of AKT are involved in other, diverse, biological functions, isoform-selective AKT-inhibitors are of particular interest.

**Figure 8:**
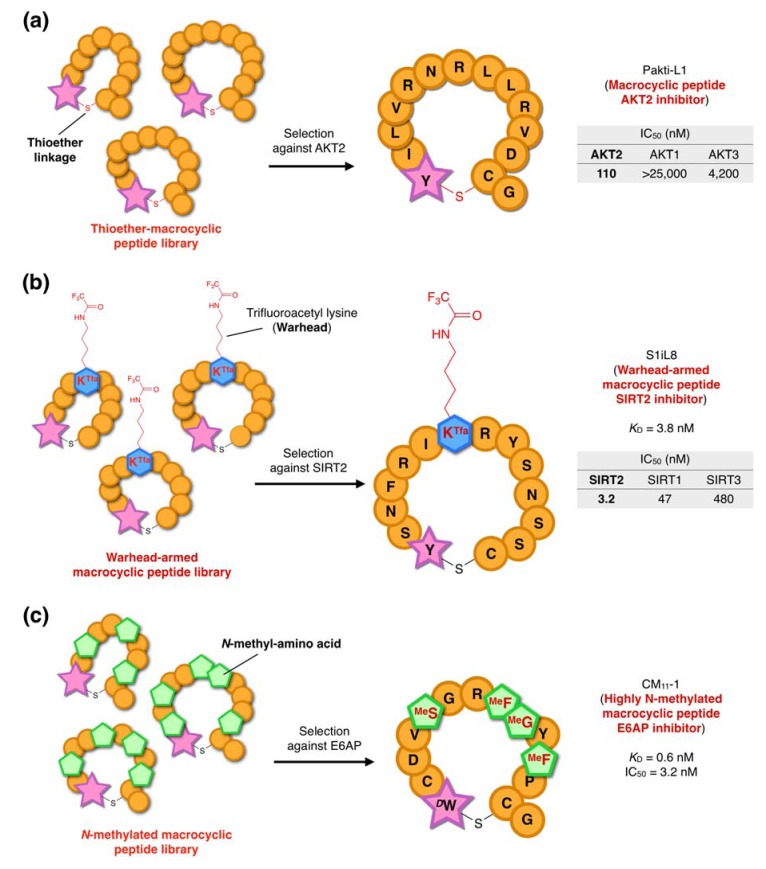
Selection of inhibitors from three distinct non-standard peptide libraries using the RaPID system. (**a**) Anti-AKT2 cyclic peptides. Thioether-macrocyclic peptide libraries were applied to the development of AKT2-selective inhibitors [60]. Pakti-L1 peptide exhibited AKT2-selective inhibitory activity with an IC_50_ of 110 nM. (**b**) Anti-SIRT2 peptides [64]. Thioether macrocyclic peptides armed with trifluoroacetyl Lys (K^Tfa^), which acts as a warhead against the SIRT2 active site. The SIRT2-selective inhibitory peptide S1iL8 is shown, which demonstrated an IC_50_ of 3.2 nM. (**c**) Anti-E6AP peptides [65]. A library of *N-*methylated thioether macrocyclic peptide library was applied to the selection against E6AP to yield several active species, one of which CM_11_-1 exhibited an IC_50_ of 3.2 nM.

Using the RaPID system, thioether-macrocyclic peptide inhibitors of AKT2 were selected from ClAc-l-Y-initiated and ClAc-d-Y-initiated libraries in parallel using full-length AKT2 immobilized on Ni^2+^-NTA magnetic beads via an N-terminal His tag (Figure 8a) [60]. These selections yielded four active l-Y-initiated thioether macrocyclic peptides (Pakti-L peptides; Pakti stands for Peptide akt inhibitor) and two d-Y-initiated peptide (Pakti-D peptides). Three Pakti-L peptides (Pakti-L1, L2, and L3) were chosen for further studies, and exhibited selective inhibition of AKT2 compared with AKT1, AKT3 and two unrelated kinases (protein kinase A and glycogen synthase kinase). The three Pakti-L peptides showed potent inhibitory activity with each exhibiting an IC_50_ of the order of 100 nM in *in vitro* assays. All demonstrated AKT2 selectivity of greater than 10-fold relative to the other two isoforms, with the most selective, Pakti-L1, exhibiting AKT2 selectivity of 250- and 40-fold to AKT1 and AKT3 respectively. This demonstrates that thioether-macrocyclic peptides derived from RaPID screening can be both potent and isoform-selective kinase inhibitors.

### 5.2. SIRT2-Isoform Selective Inhibitors with a Mechanism-Based Warhead

Human sirtuin2 (SIRT2) plays an important role in cell cycle regulation by deacetylating ε-*N-*acetylated lysine (^Ac^K) residues of α-tubulin and histone H4K16 [61,62]. When SIRT2 binds to a substrate peptide/protein, the ^Ac^K sidechain is deeply inserted into the active pocket of SIRT2 and its ε-*N-*acetyl group is hydrolyzed with consumption of NAD^+^.

A trifluoroacetyl group on the ε-amino group of lysine (K^Tfa^) has been shown to be an effective warhead for the generation of peptide inhibitors of the SIRT family, but these have generally not shown isoform-selectivity (the human SIRT family comprises three isoforms) [63]. Because the substrate binding site of SIRTs is relatively shallow, except for the ε-*N-*acetyl group binding core which is nearly identical among isoforms, rational design of isoform-selective inhibitors has proved difficult. We approached this problem using a genetic code reprogramming strategy in which the AUG codon was assigned to ClAc-l/d-Tyr as an initiator and the K^Tfa^ warhead was used as an elongator by charging them onto initiator tRNA^fMet^_CAU_ and elongator tRNA^Asn−E1^_CAU_, respectively [64] (Figure 8b). To designate the incorporation of the single K^Tfa^ at a specific position, an mRNA library was constructed in which a single internal AUG codon (for incorporation of K^Tfa^) was flanked with NNC repeats (in addition to the initiator AUG and downstream Cys codon to produce the thioether macrocycle). Selections using a ClAc-l-Tyr initiated library and a ClAc-d-Tyr initiated library were carried out in parallel, yielding 21 and 16 active clones, respectively, with a range of inhibitory activities (estimated IC_50_ values ranging from 1 nM to 100 nM).

Two representative peptides, S2iL1 and S2iD7 isolated from each library, were chosen for further studies and chemically synthesized. Surface plasmon resonance (SPR) and biochemical studies were performed to determine the kinetic and equilibrium constants (*k*_a_, *k*_d_, and *K*_D_) and the IC_50_ values. Both peptides were found to be potent inhibitors of SIRT2 with both exhibiting *K*_D_ and IC_50_ values of the order of 3 nM (note that since the determined *K*_D_ and IC_50_ values are essentially identical they are very likely equivalent to *K*_i_). Importantly, both peptides exhibited SIRT2-isoform selective inhibition with inhibitory activities against SIRT1 and SIRT3 approximately 10 and 100-fold lower, respectively. This again demonstrates the power of the RaPID system for the isolation of highly selective inhibitory peptides.

### 5.3. Thioether-macrocyclic N-methyl-peptide inhibitors against ubiquitin ligase E6AP

*N-*methylations of the peptide backbone are expected to confer drug-like properties to macrocyclic peptides, including high affinity to target proteins, elevated proteolytic resistance, and potentially cell-membrane permeability [4,5,6,66]. Since we had demonstrated the feasibility of expressing *N-*methylated macrocyclic peptides using the FIT system, we also investigated RaPID selection using such a library. For this selection, ubiquitin ligase E6AP (E6-associated protein), a member of E3 ubiquitin ligase family, was chosen as the target. E6AP promotes the degradation of the P53 tumor suppressor protein in a human papillomavirus E6 protein-dependent manner, and the HHR23A (a human homologue of the yeast DNA repair protein Rad23) and PML [67,68] tumor suppressors in an E6-independent manner. E6AP inhibitors may, therefore, have oncology applications [69].

To construct a thioether-macrocyclic *N-*methyl-peptide library, five codons were reprogrammed in the genetic code, with ClAc-d-tryptophan assigned to the initiator codon and four *N-*methyl amino acids (*N-*methylphenylalanine, *N-*methylserine, *N-*methylglycine and *N-*methylalanine; ^Me^F, ^Me^S, ^Me^G, and ^Me^A, respectively) assigned to elongator codons [65] (Figure 8c). The random sequence region was designed to have 8–15 NNU repeats, which reduced the available elongator codons to 16 (4 × 4) residues. In this system, an *N-*methyl-amino acid is expected to occur once in every four residues. After six rounds of stringent binder selection against the HECT (Homologous to E6AP C-terminus) domain of E6AP immobilized on magnetic beads, eight independent sequence families were identified. Among these were three highly abundant peptides: CM_11_-1, CM_11_-3, and CM_11_-5. It should be noted that each of these peptides contains a total of five secondary amino acids (including Pro) in the 14 peptide bonds, and therefore more than one third of the peptide bonds are alkylated. These peptides, including the designated *N-*methyl-amino acids, were chemically synthesized and further characterized in SPR assays. All exhibited *K*_D_ values of 1 nM or less, and no binding to the HECT domain of SMURF2 (an E6AP homologue) was observed. Linearized or non-methylated derivatives exhibited an almost complete loss of binding activity relative to the parental peptides, and were also found to be substantially less resistant to serum proteases.

The most potent binder, CM_11_-1, was chosen for biochemical studies. It was found that CM_11_-1 inhibited both the E6-dependent ubiquitination of P53 and the E6-independent ubiquitination of Peroxiredoxin 1. The mechanism of this inhibition appeared to be inhibition of ubiquitin-transfer from the ubiquitin donor proton E2 to E6AP, suggesting that CM_11_-1 may interfere with the E2/E6AP protein-protein interaction; an intriguing prospect since inhibition of this sort is impossible with traditional small molecule drugs.

## 6. Other Methodologies for the Selection of Non-Standard Peptides

### 6.1. Expression of Non-Standard Peptides in a Reconstituted in Vitro Translation System via *in Situ* Mischarging of tRNAs by Wildtype ARSs

An alternative technique to the FIT system for the ribosomal synthesis of non-standard peptides has been developed in parallel by the Szostak group. In this methodology, wildtype ARSs are used to mischarge amino acids with non-canonical sidechains onto endogenous cognate tRNAs in the PURE system reaction lacking some (up to 12) proteinogenic amino acids [70]. Prior to selection experiments, this research group made a remarkable effort to collate a list of non-canonical amino acids that can be mischarged onto tRNAs by ARSs and subsequently incorporated into peptide chains [71,72]. Mostly, the structures of these amino acids are closely analogous to their cognate proteinogenic amino acids, however some of the non-canonical sidechains amenable to this approach (e.g*.*, halo-aryl, alkene, alkyne, azide, and *tert*-butyl groups) are quite interesting moieties which imbue drug- or probe-like properties on the synthesized peptides.

Although this methodology is not as robust as the FIT system in terms of the range of non-proteinogenic amino acids available, it is technically simpler; a particular advantage being that commercially available amino acids can be charged *in situ* onto endogenous tRNAs in the PURE system. As discussed with respect to the FIT system above, this methodology has also been used to incorporate some *N-*methyl-amino acids into peptides [72]. However, because *N-*methyl-amino acids are generally poor substrates for wildtype ARSs, the desired *N-*methyl-aminoacyl-tRNAs were prepared not *in situ*, but via a multi-step procedure as follows: (1) ARS-catalyzed aminoacylation of the cognate tRNAs, (2) protection of the α-amino group with 2-nitrobenzaldehyde followed by reduction, (3) *N-*methylation by formaldehyde followed by reduction, and (4) deprotection of 2-nitrobenzyl group by UV irradiation. In general, observations of *N-*methyl-amino acid compatibility with the ribosomal translation obtained using the system described above have been consistent with those obtained using the FIT system. However, a notable discrepancy exists in the cases of *N-*methylated valine and leucine [73] which were reported to be efficiently elongated by the Szostak methodology but not the FIT system methodology. This issue will need to be addressed in future studies.

Two macrocyclization methodologies have also been developed for use in the PURE system. First, the sulfhydryl sidechains of two Cys residues in a peptide chain can be chemically modified by treatment with dibromoxylene to form a xylene-dithioether linked macrocycle. This chemical macrocyclization is simple and can be readily integrated with the Szostak methodology for the expression of non-standard peptides. One potential drawback compared with thioether-macrocyclization using the FIT system is that undesired or unpredictable side reactions may occur (e.g., dibromoxylene may react with residual thiol reagents in the translation buffer to generate linear peptides or, alternatively, in sequences with more than two Cys residues bromoxylene may react unselectively to yield multiple products); although it should be noted that these issues have not been fully documented. The second reported strategy is the use of 4-selenalysine (a selenocystein-derived lysine analog that can be mischarged onto tRNA^Lys^ by LysRS). Upon incorporation of this residue, the seleno group is oxidatively eliminated to yield dehydroalanine (Dha) and the sulfhydryl group at a downstream Cys generated under reducing conditions intramolecularly reacts with Dha to yield a lanthionine group. This clever thioether formation is particularly beneficial for the production of small thioether macrocyclic peptides resembling lantipeptides. An alternative approach generating a lantipeptide-like scaffold has been also reported by the Suga group [74]. In this methodology, vinylglycine is incorporated into peptide chain at a specific position by the genetic code reprograming, and isomerizes into dehydrobutyrine. This residue further reacts with a downstream Cys to yield macrocyclic peptides containing a methyllanthionine residue. Although this approach does not need external reagents for the conversion, the macrocyclization to form the methyllanthionine requires an elevated temperature.

**Figure 9:**
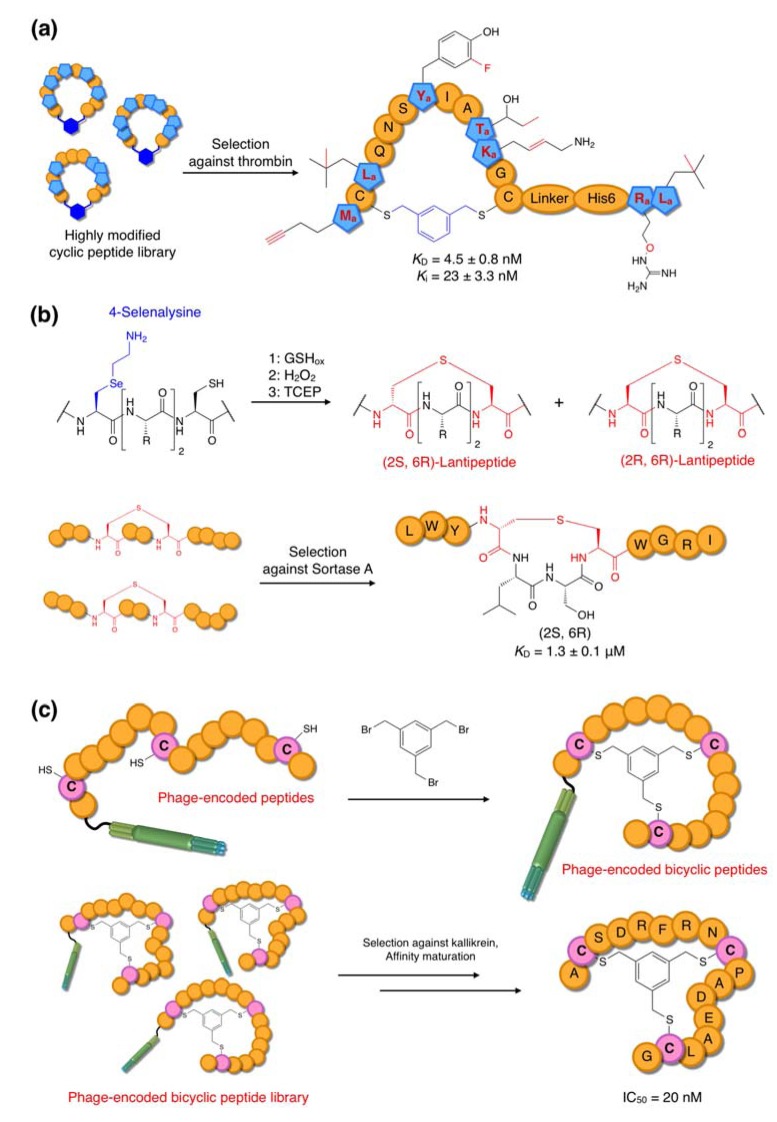
Other selection platforms. (**a**) Anti-thrombin cyclic peptides [70]. A library containing non-standard cyclic peptides containing up to 12 non-canonical amino acids was used for the selection of thrombin inhibitors. Macrocycles were formed by the reaction of two Cys residues with a bifunctional dibromoxylene linker. A selected active peptide contains six different non-canonical amino acids. (**b**) Anti-sortase A lantipeptide-like peptides [75]. An incorporated 4-selenalysine underwent elimination to dihydroalanine by H_2_O_2_ oxidation, and the lanthionine moiety was then formed by 1,4-addition of a Cys sulfhydryl group with dihydroalanine. (**c**) Anti-kallikrein “bicycle” peptides [76]. A library of peptides containing three spaced Cys residues was displayed on phage, and its treatment with TBMB yielded bicyclic peptides with six random amino acids in each loop.

### 6.2. Selection of Bioactive Non-Standard Peptides and Lantipeptide-like Peptides

The Szostak group has reported mRNA display selection of thrombin inhibitors from a xylene-dithioether-macrocyclic peptide library containing 12 non-proteinogenic amino acids in the reprogrammed genetic code [70] (Figure 9a). Thrombin is a well validated target for therapeutic intervention during thrombosis. Eight peptide families were identified after ten rounds of selection with competitive elution by hirudin, a small proteinogenic competitive inhibitor of thrombin. Upon screening of inhibitory active peptides, two peptides (the 27-mer peptides U1 and U2 containing six non-canonical amino acids each) were identified as potent inhibitors. The U1 peptide was shown to be particularly potent, exhibiting *K*_D_ and *K*_i_ values of 4.5 and of 23 nM, respectively. 

Lantipeptides are naturally occurring thioether-macrocyclic peptides generally produced by Gram-positive bacteria and, like other naturally occuring cyclic peptides, often show antibacterial activities [77]. Although many artificial lantipeptides have been synthesized and tested for antibacterial activities [78,79,80], such a semi-rational approach is not ideal for the discovery of new scaffolds. To overcome this barrier, the Szostak group used the aforementioned method involving thioether-macrocyclization via 4-selenalysine and Cys residues [75,81]. The canonical lysine (Lys) was substituted with 4-selenalysine in the Lys-deficient translation system and a lantipeptide-like peptide library was generated by the procedure discussed above (Figure 9b). Sortase A, which is a transamidase of *Staphylococcus aureus* involved in the growth of bacterial cell walls, was chosen for the selection target, since this enzyme family is widely conserved in gram-positive bacteria and inhibitors potentially possess antibacterial activity [82]. Five rounds of selection against biotinylated sortase A immobilized on streptavidin-coated beads yielded two major peptide families sharing a Leu-Trp dipeptide sequence in the *N*-terminal random region. Determination of the exact structure of each of these peptides was complicated by the potential for multiple stereoisomers of the lanthionine group. When chemically synthesized with defined stereochemistries, it was shown that one peptide, referred to as 2_(2*S*, 6*R*)_ (exhibiting *S* and *R* stereochemistry with respect to the Dha and Cys residues respectively) was responsible for the binding activity (*K*_D_ = 3 μM) whereas the other three stereoisomers, 2_(2*S*, 6*S*)_, 2_(2*R*, 6*S*)_ and 2_(2*R*, 6*R*)_, had binding affinities at least two orders of magnitude lower. Interestingly, this 2S, 6R conformation is the most abundant stereoisomer in naturally occuring lantipeptides.

### 6.3. Selection of Macrocyclic Peptides Cross-Linked via Two Amide Bonds

The Roberts group has described a methodology for the synthesis of macrocyclic peptides using a rabbit reticulocyte lysate *in vitro* translation system. In this system the N-terminus of the peptide (unlike *E. coli* based *in vitro* translation systems this eukaryotic system initiates translation from unformylated α-amino Met) is chemically cross-linked to the ε-amino group on the sidechain of Lys using disuccinimidyl glutarate (DSG) to form a propyl amide linkage. This methodology was combined with amber codon (UAG) suppression by *N-*methyl-Phe (charged onto a suppressor tRNA by classical semi-enzymatic synthesis) and an NNK library to produce cyclic peptides with a random distribution of *N-*methyl-Phe residues [83,84].

This peptide library was used for selection experiments using an mRNA display strategy. The target protein in this instance was Gαi1, an intracellular mediator of G-protein coupled receptor (GPCR) signaling that has been implicated in a range of human diseases [85]. Selection for Gαi1 binding yielded a single family of peptides with high sequence conservation. Notably, no peptides containing *N-*methyl-Phe were identified. The authors attributed this to the fact that the conserved region of the peptide sequences contained only a single Phe residue, positioned at a site structurally incompatible with *N-*methylation (an α-helical region) so that the incorporation of *N-*methyl-Phe at this position was prohibited. However, since amber codon suppression competes with translation termination, an alternative hypothesis is that poor incorporation of *N-*methyl-Phe caused transcripts containing the amber codon to give rise to truncated peptides, rather than peptides incorporating *N-*methyl-Phe. This hypothesis is in some way supported by the observation that *N-*methyl-Phe residues were also not found in the non-conserved regions of the peptides identified. Nevertheless, one of the selected cyclic peptides was shown to have a high affinity for Gαi1 (*K*_D_ ~ 2.1 nM), demonstrating the feasibility of this translation system for non-reducible, post-translationally cyclized peptide synthesis.

### 6.4. Selection of Phage-Encoded Bicyclic Peptides

Phage display is a classic technique for the isolation of peptide ligands from standard libraries. However, its use for the display of macrocyclic peptides has been limited by its exclusive reliance on the 20 canonical amino acids, meaning that cyclization was only achievable through Cys-Cys reducible disulfide bonds. More recently, the Winter group has described a methodology in which three Cys residues at specified positions in linear phage-expressed peptides are reacted with tris(bromomethyl)benzene (TBMB) to yield bicyclic peptide products, referred to as “bicycles” [76] (Figure 9c). This methodology has been applied to the identification of inhibitors of human plasma kallikrein and cathepsin G using a phage panning technique. Although the initial hits against plasma kallikrein were not particularly potent (IC_50_ ~ 1 μM), more stringent conditions were applied to a semi-randomized library derived from the initial screening results to yield stronger inhibitors with IC_50_ values in the sub-50 nM range.

## 7. Conclusions

In this review, we have discussed the utility of *in vitro* selection systems (the RaPID system and other selection systems) for the isolation of peptide ligands to various protein targets of therapeutic interest. These systems have great promise as novel drug discovery technologies since they enable the construction and screening of highly diverse peptidomimetic or natural product-inspired peptide libraries of a trillion different compounds, allowing for the expeditious and low-cost discovery of bioactive molecules. Recently obtained X-ray structures of some thioether-macrocyclic peptides bound to targets (unpublished results) have revealed that they appear to have much greater areas of target interaction (involving specific hydrogen bonding and hydrophobic interactions) than small organic molecules. This raises the possibility that such macrocyclic peptides may be viable inhibitors of so-called “non-drugable” targets, by interfering with both extracellular and intracellular protein-protein interactions. In fact, many of the technologies discussed in this review are already being implemented in drug discovery programs in the biotech industry. The RaPID system itself is currently being improved to widen the scope of potential targets and to incorporate a wider range of non-standard scaffolds. These emerging technologies will enable us to open new avenues of research into mid-sized, natural product-inspired peptides or peptidomimetics that have been modestly investigated in the past. 
